# Microbiota-mitochondria axis in neurodegenerative disorders and retinal dysfunction: mechanisms and emerging therapeutic opportunities

**DOI:** 10.3389/fphar.2026.1889898

**Published:** 2026-09-01

**Authors:** Puneet K. Samaiya, Akshat Agrawal, Shrija Kesharwani, Rini Sathe, Santosh Kumar Prajapati, Shreyasi Majumdar

**Affiliations:** 1 Department of Pharmacy, Shri G.S.Institute of Technology & Science, Indore, Madhya Pradesh, India; 2 Department of Neurosurgery Brain and Spine, University of South Florida, Tampa, FL, United States; 3 Department of Pharmaceutical Technology, School of Health and Medical Sciences, Adamas University, Kolkata, India

**Keywords:** antibiotic resistance, gut dysbiosis, gut microbiome, metabolic disease, neurodegenerative disease, neuroinflammation

## Abstract

The gut microbiota crucial role in maintaining host metabolism, immune homeostasis, intestinal integrity, and mitochondrial function. Emerging evidence suggests that dysregulation of the microbiota–mitochondria axis represents one of the key mechanisms linking gut dysbiosis to the development of neurodegenerative and ocular diseases. The dysbiosis causes disruptions in mitochondrial physiology due to lower levels of short-chain fatty acids, increased intestinal permeability, endotoxemia, oxidative stress, and inflammation. These mechanisms contribute to the pathogenesis of Alzheimer’s disease, Parkinson’s disease, age-related macular degeneration, diabetic retinopathy, retinitis pigmentosa, and central serous chorioretinopathy. Environmental factors such as unhealthy dietary patterns, excessive antibiotic use, and prolonged exposure to artificial blue light may exacerbate dysbiosis and further disrupt mitochondrial homeostasis, thereby contributing to disease progression. We also elaborated the emerging therapeutic strategies targeting both the gut microbiota and mitochondrial function, including next-generation probiotics, postbiotics, dietary interventions, mitochondria-targeted antioxidants, NAD^+^ precursors, and precision medicine approaches. Collectively, current evidence represents the microbiota–mitochondria axis as a one of the promising mechanistic and therapeutic targets for neurodegenerative and ocular diseases. However, most therapeutic evidence discussed in this review is derived from preclinical studies, with relatively limited clinical validation. Future well-designed longitudinal studies and randomized clinical trials are essential to establish efficacy, safety, and translational applicability in humans.

## 1 Introduction

The gastrointestinal tract (gut) harbours more than 100 trillion complex microorganisms (including bacteria, fungi, protists, archaea, and viruses) and, in some areas, up to a density of 1,012 bacteria per millilitre. This gut microbiome is the hidden key to health. Together, they create a micro-ecosystem that interacts with the larger ecosystem of the human body (Scuderi et al., 2022; Sender et al., 2016). Interestingly the human genetic blueprint holds around 23,000 genes, whereas the gut microbiome has more than three million. Therefore, the metabolic byproducts produced from the genes of the microbial DNA can affect human health (Qin et al., 2010). Microbial density and composition vary along the gastrointestinal tract, with relatively fewer microbes in the upper intestine and a higher abundance of predominantly anaerobic bacteria in the colon, where oxygen availability is low. Different parts of the GI tract have different conditions, such as pH, oxygen levels, and food availability; these conditions affect what kinds of microbes live there (Donaldson et al., 2016). It is widely recognised that the gut microbiome imparts an indispensable role in digestion, immunity, and virtually all aspects of health. These microbes cooperate with the human body in a symbiotic relationship. Such a cooperation helps control important bodily functions, especially those of the immune and metabolic systems. They also strengthen the intestinal barrier to prevent harmful substance leakage into the body. This is achieved by microbes located in the gut, breaking complex carbohydrates to mean, fibres or things our body cannot break down on its own. While some of the microbes are more abundant than others, even low-abundant species can have large effects on health. Thus, it is not only about which bacteria are present (taxonomy) but also what function they perform (functional features). Researchers study these functions by investigating the genes, proteins, and pathways involved in the microbiome’s activity. This can identify targets for therapies aiming at changing microbiomes for the better. It is known that current medicine can now consider only the potential impact of an imbalance or balance of microbiome on health-related conditions, including gastrointestinal disease ocular toxicity and mental health. To create new therapy approaches in relation to maintaining a healthy equilibrium of gut microbes, researchers should know how the microbiome works and its relation to the human body (Gilbert et al., 2018). The gut microbiota plays a vital role in fermenting of non-digestible substrates like dietary fibres and endogenous intestinal mucus. This process of fermentation promotes the proliferation of specific microbes that produce Short Chain Fatty Acid (SCFA) and gases while butyrate, propionate and acetate are byproducts of this process. SCFAs are essential since they defend against bad bacteria and reduce the inflammation in the gut (Koh et al., 2016; Prajapati et al., 2025a). Butyrate acts as the main energy source for colonocytes, promotes programmed cell death in colon cancer cells, and supports intestinal gluconeogenesis, which aids glucose and energy balance. It also enables oxygen utilization through β-oxidation in the epithelial cells, to prevent microbiota imbalance. Propionate influences the function of the liver gluconeogenesis and appetite signals by interacting with the fatty acid receptors in the gut. Acetate, the most abundant SCFA, promotes the growth of bacteria, cholesterol metabolism and lipogenesis, and has effects on appetite regulation. Increased SCFA production is related to reduced diet-induced obesity and insulin resistance. Although butyrate and propionate have effects on gut hormones and appetite suppression in mice, acetate lacks these effects. Gut microbiota enzymes metabolize bile acids and form molecules that regulate important pathways in the host (Louis and Flint, 2017).

**FIGURE 1:**
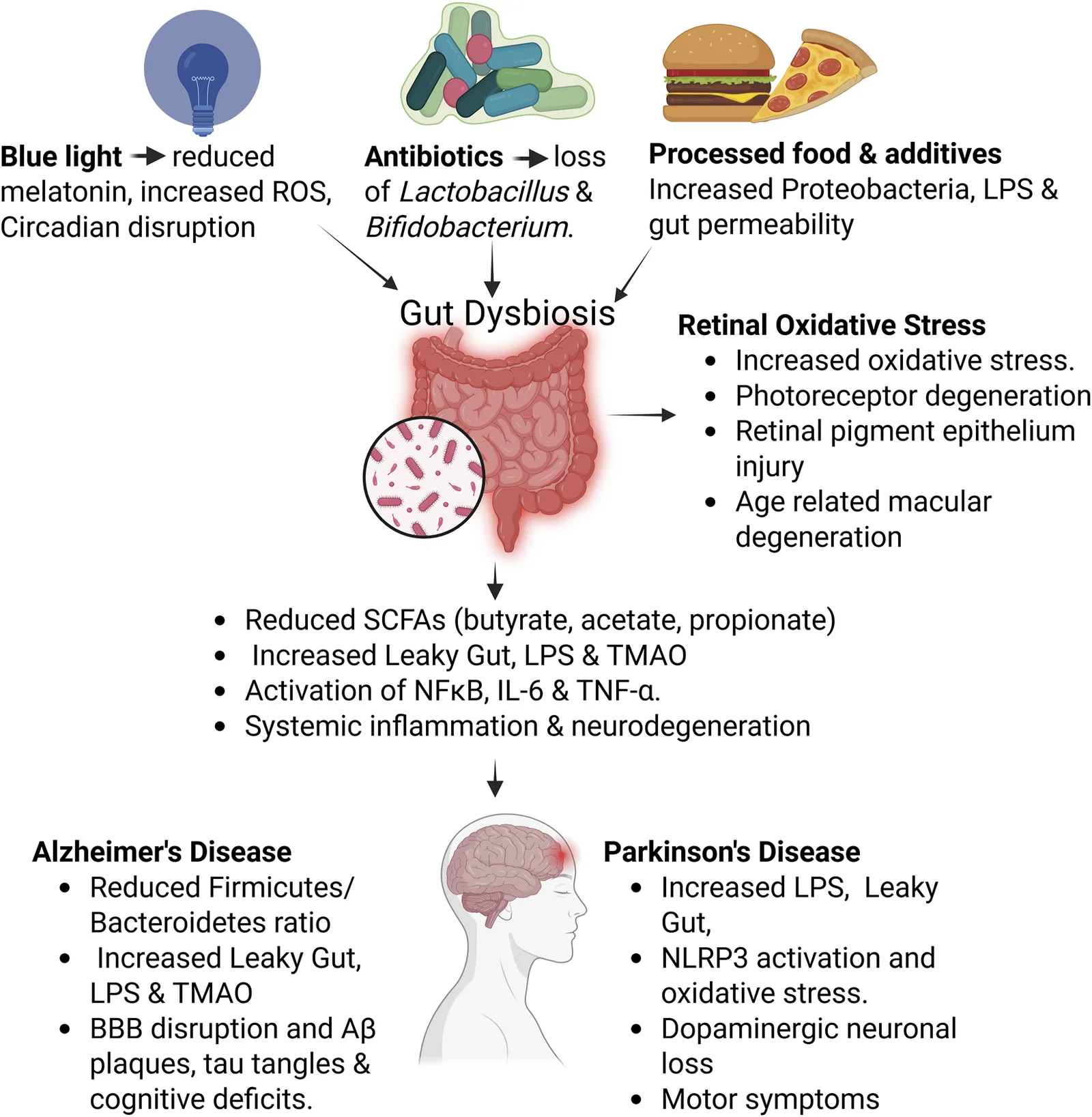
The gut–retina–brain axis in neurodegeneration. Blue light exposure, antibiotic use, and processed food/additives each drive gut dysbiosis, characterized by loss of beneficial bacteria (*Lactobacillus, Bifidobacterium*), increased Proteobacteria, and elevated LPS and gut permeability. This dysbiosis reduces protective short-chain fatty acids while raising circulating LPS/TMAO and pro-inflammatory signaling (NF-κB, IL-6, TNF-α), leading to retinal oxidative stress and photoreceptor damage (contributing to age-related macular degeneration) as well as systemic neuroinflammation that promotes Alzheimer’s disease (amyloid-β/tau pathology, blood-brain barrier disruption) and Parkinson’s disease (NLRP3 activation, dopaminergic neuron loss).

Several studies suggest that mitochondrial dysfunction acts as an important mechanism connecting gut dysbiosis to neurodegeneration and retinal disorders (Cryan et al., 2019; Fang et al., 2020). Alterations in gut microbial composition influence host mitochondrial function through microbial metabolites, inflammatory mediators, oxidative stress pathways, and immune signaling cascades (Dalile et al., 2019; Agus et al., 2018). These mechanisms coordinate the functioning of mitochondrial bioenergetics, the generation of reactive oxygen species, mitophagy, and neuronal survival (Murphy, 2009; Pickles et al., 2018; Chan, 2020). As a result, a new concept of the microbiota-mitochondria-neuron axis emerges, which offers an integrative view of how alterations in the microbial balance in the gut lead to the development of such diseases as Alzheimer’s, Parkinson’s, and retinal degeneration (Fang et al., 2020; Lautrup et al., 2019) (Figure 1). This review therefore elaborating mitochondrial dysfunction as a pathological cause connecting gut dysbiosis with neural injury (Lautrup et al., 2019; D’Amico et al., 2021). Further, this review emphasises emerging mitochondria-targeted therapeutic strategies that may restore microbial and neuronal homeostasis.

## 2 Literature search strategy

The narrative review was carried out through the systematic search of literature in the PubMed, Scopus, Web of Science, and Google Scholar databases. The search entailed articles published until January 2025 by combining different keywords including: “gut microbiota,” “gut dysbiosis,” “microbiota-gut-brain axis,” “microbiota-mitochondria axis,” “mitochondrial dysfunction,” “mitophagy,” “oxidative stress,” “Alzheimer’s disease,” “Parkinson’s disease,” “retinal diseases,” “age-related macular degeneration,” “diabetic retinopathy,” “MitoQ,” “Elamipretide,” “SS-31,” “coenzyme Q10,” “nicotinamide riboside,” “urolithin A,” “probiotics,” and “postbiotics.” The references in the relevant articles were also manually searched for more articles. Original peer-reviewed research articles, systematic reviews, meta-analyses, randomized controlled trials, Mendelian randomization studies, and other high quality experimental studies pertaining to the microbiota-mitochondria axis received top priority. Non-English language articles, conference abstracts, editorials, commentaries, duplicates, and insufficiently documented studies were excluded from the review. The literature was critically appraised and analyzed to present a state-of-the-art review on the potential mechanism and therapeutic implications of the microbiota-mitochondria axis in neurodegenerative and retinal diseases.

## 3 Gut dysbiosis and its correlation to leaky gut

The gut microbiome plays a key role in host metabolism, immune system, and barrier function maintenance (O'Hara and Shanahan, 2006). Dysbiosis, a disruption of the balanced microbial environment within the host body, is increasingly being linked to various diseases, such as infections, inflammation, metabolism, neurodegeneration, and ophthalmological disorder (Bidell et al., 2022). The characteristics of the gut dysbiosis condition include changes in the microbiome’s composition and functional abilities leading to a reduction in the level of diversity and imbalance of beneficial and pathogenic microbe (Thursby and Juge, 2017; Verma et al., 2021). According to the Anna Karenina principle, each dysbiotic microbiome exhibits a unique pattern of microbial disruption rather than a uniform disease-associated signature (Ke et al., 2021; McBurney et al., 2019). However, one of the most common signatures of the dysbiosis condition is the expansion of *Proteobacteria* species, especially *Escherichia coli* and *Klebsiella* spp., whose levels may increase from less than 10% in healthy individuals to 20%–30% in affected subjects (Chang et al., 2008; Shahinas et al., 2012; Sorbara and Pamer, 2022; Rinninella et al., 2019). The dysbiosis condition may be caused by various external factors, such as blue light exposure, unhealthy diet, drinking alcohol, infections, and antibiotics intake (Stolfi et al., 2022). Furthermore, there are some non-antibiotic medications that may affect the gut microbial composition through decreasing the number of beneficial and increasing the amount of potentially pathogenic microbes (Bruno et al., 2019; Sjöstedt et al., 2021). A summary of the major beneficial and detrimental gut microorganisms and their functional roles is provided in Table 1.

**TABLE 1:** Beneficial and detrimental bacteria in human gut.

Bacteria name	Metabolites/Products	Mechanism	Associated diseases/Conditions	References
*Lactobacillus*	Lactate, SCFAs	Supports mitochondrial respiration, reduces oxidative stress, strengthens intestinal barrier	AD, PD, retinal disorders	(Liu et al., 2018)
*Bifidobacterium*	Acetate, GABA	Anti-inflammatory effects, improves mitochondrial energy metabolism and gut barrier integrity	AD, metabolic disorders	(O’callaghan and Van Sinderen, 2016; Parker et al., 2020)
*Faecalibacterium*	Butyrate	Enhances mitochondrial biogenesis, activates AMPK-PGC1α pathway, reduces neuroinflammation	AD, Stroke	(Dalile et al., 2019; Miquel et al., 2013)
*Akkermansia muciniphila*	Acetate, propionate	Maintains mucosal barrier, improves mitochondrial metabolic homeostasis	Obesity, retinal diseases	(Cani and de Vos, 2017; Depommier et al., 2019)
*Roseburia*	Butyrate	Promotes oxidative phosphorylation and anti-inflammatory signaling	AD, PD	(Tamanai-Shacoori et al., 2017)
*Escherichia/Shigella*	LPS	Induces ROS production, mitochondrial dysfunction, neuroinflammation	AD, PD	(Fang et al., 2020; Cattaneo et al., 2017)
*Clostridioides difficile*	Toxins A/B	Disrupts epithelial barrier, induces mitochondrial stress and inflammation	Gut-brain disorders	(Abt et al., 2016)
*Enterobacteriaceae*	LPS, endotoxins	mtDNA damage, oxidative stress, chronic inflammation	Stroke, neurodegeneration	(Xu et al., 2021)

Dysbiosis can also modulate the pharmacodynamics and pharmacokinetics of various medications. For instance, certain gut microbes can bio-transform tacrolimus into less active derivatives, potentially explaining interindividual variability in drug bioavailability (Guo et al., 2019). Similarly, the efficacy of metformin has been linked to specific commensals, as its glucose-lowering effects are significantly diminished when co-administered with oral vancomycin. The cardiac glycoside digoxin is rendered inactive by *Eggerthella lenta*, and the effectiveness of warfarin, vitamin K antagonists may be augmented in patients whose microbiota-mediated vitamin K production is impaired by antibiotic use (Kim et al., 2021; Marchesi et al., 2016). Furthermore, the therapeutic impact of immune checkpoint inhibitors on tumour progression may be enhanced through the reintroduction of certain commensal strains (Rani et al., 2021; Camelo-Castillo et al., 2021). Following dysbiosis, restoration of the gut microbiome to its pre-disturbance state may occur spontaneously with the cessation of the initiating factor (e.g., an antibiotic) or be facilitated through targeted interventions. Using prebiotics and probiotics are among the more accessible strategies. Prebiotics function as substrates that nourish beneficial gut bacteria, including butyrate producers, while probiotics are described as “live microorganisms which when administered in adequate amounts confer a health benefit to the host” and are meant to actively support and re-establish microbial balance. A more sophisticated and resource-demanding option is microbiota-based transplantation, typically performed using stool samples from healthy donors (Su et al., 2020). It may result from host-related factors such as genetic predisposition, existing health conditions (e.g., infections, inflammation), and lifestyle behaviours, but is more prominently influenced by environmental elements such as dietary patterns (particularly excessive sugar and lake of fibre intake), as well as contact to xenobiotics (including antibiotics, pharmaceuticals, and food additives), and sanitation habits (Hrncir, 2022).

Dietary modifications have beneficial physiological outcomes; for instance, consumption of diets rich in simple sugars compromise the intestinal lining, incite intestinal inflammation, and adversely affect metabolic regulation. However, these detrimental effects often require the presence of the microbiota to manifest, as they are typically absent in germ-free animal models. Moreover, the transfer of dysbiotic microbiota through fecal transplantation has been demonstrated to reproduce disease phenotypes in recipients (Vrieze et al., 2012; Schulz et al., 2014; Collins et al., 2013).

Although historically unrecognized, the result of food additives on the gut microbiota has been recently garnered attention. Emerging research indicates that certain human gut microbes exhibit marked sensitivity to commonly used preservatives (Hrncirova et al., 2019a), and that exposure to these compounds can encourage the proliferation of *Proteobacteria* (Hrncirova et al., 2019b). Various classes of additives have also shown deleterious outcomes. For instance, dietary emulsifiers have been found to disturb the profile of gut microbial communities and provoke intestinal inflammation (Chassaing et al., 2017). Similarly, artificial sweeteners such as Splenda have been associated with *Proteobacteria* overgrowth and have been shown to worsen ileitis in SAMP1/YitFc (SAMP) mice. Ironically, calorie deficient synthetic sweeteners were initially presented to mitigate metabolic disorders; however, they have been found to trigger dysbiosis and enhance glucose intolerance in a microbiota-dependent manner, thereby contributing to the very metabolic disbalances which they were intended to prevent (Rodriguez-Palacios et al., 2018).

Intestinal wall integrity may be impaired due to acetaldehyde generated from ethanol either endogenous or exogenous via microbial metabolism, through direct mucolytic enzymatic activity (Llorente and Schnabl, 2015), and by other biochemical pathways. The immune system is also affected by microbial metabolites and structural elements that activates inflammasomes or engage Toll-like receptor (TLR) and NOD-like receptor (NLR) pathways. Furthermore, dysbiosis can disrupt the equilibrium between regulatory and pro-inflammatory immune cell populations. Metabolic disturbances, especially those involving glucose and lipid processing, may arise through alterations in bile acid profiles, reductions in SCFA production from fibre, derived from the diet, the microbial transformation of choline to trimethylamine (TMA), and various other mechanisms (Chu et al., 2019). Changes are driven by exposure to toxins, pharmaceutical agents, and infectious organisms. Among these, enteric pathogens are particularly potent in inducing microbial dysbiosis. This has been shown in various experimental studies performed on animals, where infection with food containing viral pathogens has led to both localized and systemic inflammatory responses, thereby altering microbiota composition and compromising barrier integrity of intestine.

## 4 Microbiota–mitochondria axis in neurodegenerative and ocular diseases

Findings show that gut microbes metabolites play a crucial role in the regulation of mitochondrial activity, thus serving as an important mechanism behind the connection between gut dysbiosis and neurodegeneration (Cryan et al., 2019; Fang et al., 2020). Mitochondria are responsible for energy generation, redox status regulation, calcium balance, programmed cell death, and innate immunity signaling. Therefore, the changes in gut microbiome associated with decrease in SCFA synthesis, higher systemic levels of lipopolysaccharide (LPS), disturbances in bile acid metabolism, and accumulation of metabolites like TMAO may significantly impair mitochondrial performance (Cryan et al., 2019; Parker et al., 2020).

Among the metabolites produced by microorganisms, SCFAs, such as butyrate, acetate, and propionate, play an essential role in mitochondrial bioenergetics. Butyrate is an energy source for colonocytes and enhances mitochondrial respiration through activation of AMP-activated protein kinase (AMPK) and PGC-1α, the major factor that stimulates mitochondrial biogenesis (Dalile et al., 2019; Silva et al., 2020). Reduced amounts of butyrate-producing bacteria, such as *Faecalibacterium*, *Roseburia*, and *Eubacterium*, caused by dysbiosis impair mitochondrial respiratory chain function and ATP production.

In contrast, LPS, which forms part of Gram-negative bacteria cell walls, negatively affects mitochondria. The increase in permeability of the intestinal wall due to dysbiosis facilitates LPS transfer into the bloodstream. This induces TLR4-NF-κB signaling, resulting in ETC., inhibition, mitochondrial membrane potential decline (ΔΨm), disturbance of oxidative phosphorylation and increased generation of mitochondrial reactive oxygen species (mtROS), mtDNA damage, and neuroinflammation (Fang et al., 2020; Zhang et al., 2022).

The same applies to TMAO, which is formed by microbial transformation of dietary choline and carnitine. High levels of TMAO correspond to low respiratory chain complex activity, endothelial dysfunction, oxidative stress, and NLRP3 inflammasome activation, causing neuronal damage (Vogt et al., 2018; Ji et al., 2025). Microbial tryptophan metabolism leads to the formation of indole metabolites, kynurenines, and serotonin precursors, regulating mitochondrial energy production via AhR-dependent pathways; dysbiosis-related changes in the levels of these compounds impair mitochondrial function and accelerate neurodegeneration (Agus et al., 2018; Kennedy et al., 2017).

Dysfunctional mitochondria have also gained importance in the pathogenesis of AD, PD, retinal degeneration, diabetic retinopathy, and age-related macular degeneration (López-Otín et al., 2023; Chen et al., 2023). One such mechanism is excess mtROS generation: inflammation through dysbiosis affects the efficiency of the ETC., and leads to the release of electrons from complex I and complex III along with the generation of excess amounts of superoxide and hydrogen peroxide (Murphy, 2009). This process poses a particular problem to neurons, as they have high metabolic needs, low regenerative capability, and a large amount of lipids; oxidation of proteins, lipids, and mtDNA, which is without histone protection and lacks repair mechanisms, causes impairment in ATP synthesis and apoptosis (Swerdlow, 2018). MtDNA also acts as a DAMP, leading to activation of NLRP3 inflammasome signaling (West et al., 2011).

The resulting dysbiosis-induced mitochondrial stress also results in malfunctioning of mitophagy. Regular mitochondrial fusion and fission, which is dependent on mito-fusins (MFN1/2), OPA1, and DRP1 is disturbed and causes mitochondrial fragmentation and mitochondrial bioenergetics dysfunction (Chan, 2020; Prajapati et al., 2024). The mechanism of mitophagy, that is a selective elimination of damaged mitochondria, is affected as well; mitophagy deficiency mediated by PINK1-Parkin causes accumulation of mitochondria defects, excessive ROS formation and progressive neurodegeneration in Alzheimer’s and Parkinson’s diseases (Pickles et al., 2018), as well as in retinal degeneration or ocular toxicity.

**FIGURE 2:**
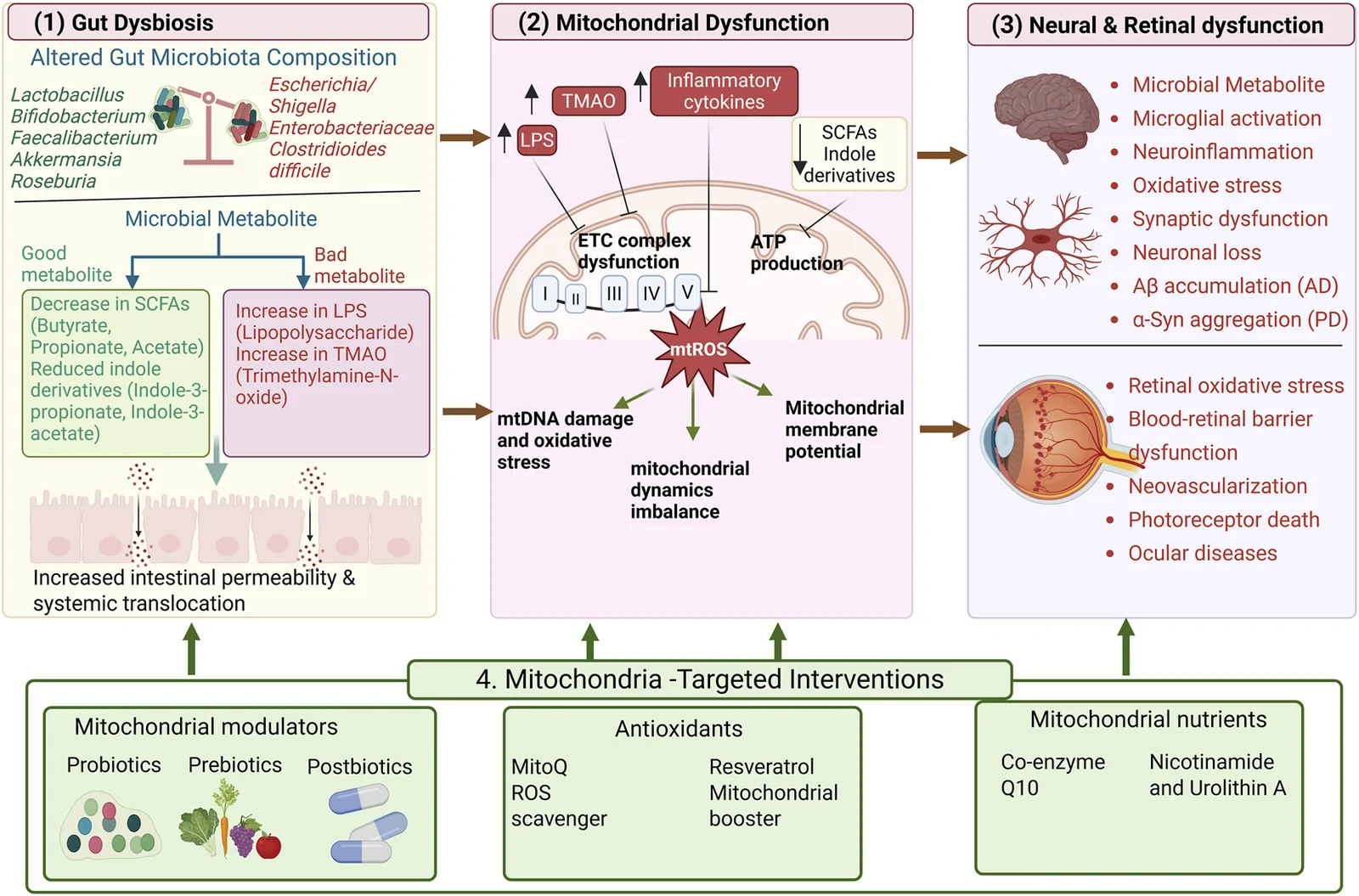
Proposed mechanistic pathway linking gut dysbiosis, mitochondrial dysfunction, and neural/retinal degeneration, with potential mitochondria-targeted therapeutic interventions. (1) Gut Dysbiosis: Changes in the gut microbiome are marked by reduction in good bacteria (*Lactobacillus*, *Bifidobacterium*, *Faecalibacterium, Akkermansia, Roseburia*) and an increase in harmful bacteria (*Escherichia/Shigella, Enterobacteriaceae, Clostridioides difficile*). This change is seen in the production of metabolites by the microorganisms, such that good metabolites are decreased (including short-chain fatty acids [SCFAs: butyrate, propionate, acetate] and indoles [indole-3-propionate, indole-3-acetate]) while bad metabolites are increased (such as lipopolysaccharide [LPS] and trimethylamine-N-oxide [TMAO]). This causes intestinal permeability dysfunction, causing systemic exposure to microbes and their products. (2) Mitochondrial Dysfunction: LPS, TMAO, inflammatory cytokines, along with reduced SCFAs and indoles, exert an effect on mitochondria resulting in ETC., complex I–V dysfunction and reduced ATP synthesis. This leads to increased production of mtROS, which in turn induces mtDNA injury, oxidative stress, dysregulation of mitochondrial dynamics (fusion/fission imbalance), and mitochondrial membrane potential loss. (3) Subsequent downstream dysfunction of mitochondria leads to microglial activation, neuroinflammation, oxidative stress, and synaptic dysfunction in the brain, resulting in neuronal cell death, accumulation of amyloid-β (Alzheimer’s disease), and accumulation of α-synuclein (Parkinson’s disease). Simultaneously, retina tissue manifests signs of oxidative stress, dysfunction of the blood-retinal barrier, development of neovascularization, and death of photoreceptors, ending up with ocular disorder. (4) Targeted Therapies for Mitochondria: The suggested therapies that target different stages of this process comprise the use of probiotics, prebiotics, postbiotics to induce eubiosis; antioxidants (MitoQ, resveratrol, ROS scavengers, and mitochondrial boosters) to alleviate oxidative stress; and mitochondrial nutrients (coenzyme Q10, nicotinamide, urolithin A) to protect mitochondria bioenergetics.

Despite the different clinical manifestations of particular neurodegenerative and ocular diseases, mitochondria dysfunction represents a common pathway involving oxidative stress, chronic neuroinflammation, respiration disturbance, mitophagy defects and metabolic disruptions (Figure 2).

### 4.1 Alzheimer disease (AD)

AD is a relentlessly progressive condition involving neuronal atrophy and is specific to the CNS, characterized by the gradual decline of cognitive functions. The pathologically identifiable signs of AD involve the accumulation of amyloid-beta (Aβ) in the form of extracellular neuritic plaques and the phosphorylation of tau protein, appearing in the form of neurofibrillary tangles (NFTs) (Long and Holtzman, 2019; Majumdar et al., 2026a). As the most prevalent subtype of dementia, has gained the reputation of being a global public health priority affecting an estimated 50 million people worldwide (Hodson, 2018). The evolving concept of the “microbiota-gut-brain axis” an elaboration of the previously used “gut-brain axis” recognizes the pivotal role of the gut microbiota in governing central neural functions. While mechanisms underlying the interactions between the gut microbiota and CNS are yet to be fully understood, there has been a body of scientific data obtained via investigations conducted on germ-free (GF) mice, application of antibiotics and probiotics showing several mechanisms of communication between the GI tract and the CNS. Neurodegenerative disorders such as PD, and AD are characterized by the gradual accumulation of abnormal protein aggregates in the CNS (Jucker and Walker, 2018). The famous claim made by Hippocrates that “all diseases start from the gut,” which was put forward more than two thousand years ago, still remains one of the major subjects of study and discussions in today’s science (Cryan et al., 2019). The alterations in the composition of the gut microbiota observed in the APP/PS1 transgenic mice have been associated with increased Aβ accumulation and cognitive deficits (Brandscheid et al., 2017; Shen et al., 2017). Nonetheless, these observations have only been shown experimentally in animal models and cannot necessarily be applied to humans. Moreover, the pathological accumulation of Aβ in myenteric neurons along with activation of intestinal innate immunity occurs before inflammation appears in the CNS of AD mouse models (Semar et al., 2013). Similarly, in the case of Tg2576 transgenic AD model mice, the disruption of the integrity of the intestinal epithelium barrier, as well as Aβ deposition in the gut vasculature, have been observed prior to the development of cerebral Aβ pathologies.

In support of this assertion, postmortem analysis has provided evidence for the formation of Aβ deposits in the intestines of patients suffering from AD, thus adding credence to the theory that Aβ aggregation in the gut is likely to predate Aβ aggregation in the brain (Honarpisheh et al., 2020). The gut microbiota has been suggested to influence mechanisms involved in Aβ transport through microbial metabolites such as TMAO. This compound stimulates platelet hyper-reactivity by promoting the intracellular release of calcium ions in response to stimulation (Figure 2) (Zhu et al., 2016). However, these findings are primarily derived from transgenic mouse models and have not yet been conclusively validated in large prospective human cohorts.

In addition to this, there has been an increasing number of evidence suggesting the possible link between AD and infections due to various microbes, as well as from fungi, *Chlamydia* pneumoniae, and spirochetes (Wozniak et al., 2009; Woods et al., 2020). Despite the fact that there is currently no cure for AD, recent findings bring hope, specifically concerning the role of the gut microbiota in AD pathology.

Though the hypothesis regarding the involvement of microbes in AD pathology is not completely new, recent research have brought some experimental evidence supporting this idea. It was found that metabolites of microbes in the CSF of AD patients were significantly correlated with several AD markers like phosphorylated tau and tau/Aβ42 ratios (Vogt et al., 2018). A study conducted on gene sequencing of 16S rRNA of faecal matter from transgenic mice expressing human Aβ precursor protein (APP), there were clear differences in terms of gut microbiome compared to the wild-type animals. In addition to this, AD model mice show distinct gut microbial fingerprints. Notably, treatment with broad-spectrum antibiotics for prolonged antibiotic treatment was associated with reduced Aβ burden in transgenic mouse models (Minter et al., 2016). Whether similar effects occur in humans remains unknown. More detailed studies in comparing SCFAs and gut microbiome profile between wild-type and AD mice during various developmental stages revealed major changes in the microbial flora. Particularly, mice that showed AD phenotype have a reduced abundance of beneficial microbes including *Butyricicoccus* and *Ruminococcus* while an increase in *Proteobacteria* and *Verrucomicrobia* was recorded (Sorboni et al., 2022).

Recent studies have revealed important associations between certain bacterial taxa and the CSF biomarkers that are related to the pathology of AD. For example, reduced values of such important CSF biomarkers as the Aβ42/Aβ40 ratio, p-tau and the p-tau/Aβ42 ratio are found to be positively correlated with the presence of *Clostridiaceae* (SMB53) and *Erysipelotrichaceae* (cc115). On the other hand, an increase in the amount of *Blautia* and *Bacteroides* bacteria was found to be correlated with elevated amounts of these CSF biomarkers, thereby creating a protective profile of bacteria (Cattaneo et al., 2017). The analysis of the results obtained by Verhaar et al. (2022) with the use of machine learning predictive model revealed several important microbial taxa associated with tau pathology. *Lachnospiraceae* bacteria, *Lachnoclostridium edouard*, and *Blautia faecis*, which belong to Firmicutes phylum, were the best predictors of the tau presence. Such model showed high predictability when used for tau prediction through the area under the curve (AUC) analysis (Verhaar et al., 2022). Dysbiosis has been found in phyla within the gut bacteria such as *Firmicutes, Proteobacteria,* and *Bacteroidetes* through both clinical studies on human subjects and experiments performed using AD transgenic animals (Hrncir, 2022). Although changes in the levels of *Actinobacteria* and *Verrucomicrobia* have been noted, these phyla are usually not prevalent in the GI tracts of people affected by AD (Dissanayaka et al., 2024). In an important study, Cattaneo et al. (2017) found that patients showing symptoms of cerebral amyloidosis have a different composition of their gut microbiota than those lacking neuropathological traits. Furthermore, in the former case, there were high levels of proinflammatory cytokines such as IL-6, CXCL2, NOD-like receptor protein 3 (NLRP3), and interleukin-1 beta (IL-1β), while in the latter case, there were low levels of the anti-inflammatory cytokine IL-10. Notably, the presence of proinflammatory cytokines was found to be positively correlated with *Escherichia/Shigella* and negatively correlated with *Eubacterium* (Cattaneo et al., 2017). While experimental studies consistently demonstrate that gut microbiota may influence amyloid pathology, findings from human cohort studies are more heterogeneous, and causal relationships remain to be established.

### 4.2 Parkinson disease (PD)

PD is the second most common form of neurodegenerative disease following Alzheimer’s disease, impacting anywhere from seven to ten million people around the world, and expected to reach two times that number by 2030. The clinical features of Parkinson’s disease involve the presence of motor symptoms such as resting tremors, rigidity, posture problems, and bradykinesia in addition to other symptoms like anosmia, insomnia, anxiety, depression, orthostatic hypotension, and GI dysfunction (Nair et al., 2018). In the former, manifestations such as dyspepsia, hypersalivation, constipation, defecatory disturbances, fecal incontinence, nausea, and abdominal pain tend to occur before the development of motor symptoms. In the case of constipation, it is seen to be an important early sign of dysfunction of the intestine, as well as one of the major clinical signs of the physiological mechanisms that precede disease development (Kalia and Lang, 2015; Huang et al., 2021). Major neuropathological features of PD involve the progressive loss of dopaminergic cells of the substantia nigra (SN) along with the development of Lewy bodies—cytoplasmic inclusion bodies consisting of misfolded, aggregated α-synuclein (αSyn). The importance of αSyn aggregates lies in the fact that these aggregates are not only restricted to the SN, but are also found in the brainstem, olfactory bulb, and the gastrointestinal tract. Due to this extensive localization, a prion-like theory for the progression of PD has been proposed, in which the mutant αSyn starts aggregation in the gut and spreads to the CNS through vagus nerve (Punsoni et al., 2019).

Intestinal dysbiosis has been proposed as a potential contributor to PD pathogenesis. Moreover, presence of gut microbiota was associated with aggravated motor dysfunction in α-Syn-overexpressing mouse models (Sampson et al., 2016). Supporting this, chronic oral administration of rotenone, a mitochondrial complex I inhibitor widely used as an environmental toxin model of PD, induces gastrointestinal dysfunction and marked alterations in gut microbial composition prior to the onset of motor symptoms and central nervous system pathology (Yang et al., 2017). Although these experimental observations provide mechanistic support for microbiota-mediated modulation of Parkinsonian pathology, direct causal evidence in humans remains limited.

Increasing numbers of clinical studies demonstrate dysbiosis of the intestine in PD patients when compared to healthy controls, in which changes are noted in the composition of microbes in both feces and mucosa, in families, genera, and operational taxonomic units (OTUs). Healthy controls exhibit higher levels of butyrate-producing species in their feces than do PD patients. Genomically, the microbiome associated with PD is characterized by reduced gene expression for metabolic functions and increased expression of genes for the production of LPS (Keshavarzian et al., 2015).

SCFAs, mainly acetate, propionate, and butyrate, are key metabolites produced during bacterial fermentation. PD patients exhibit highly deficient numbers of bacteria that produce SCFAs, including *Lachnospiraceae* (Hill-Burns et al., 2017; Jin et al., 2019) and *Faecalibacterium*, along with decreased concentrations of fecal SCFAs (Perez-Pardo et al., 2018). Butyrate, the primary fuel for colonocytes, is almost completely metabolized locally, and decreased amounts of butyrate can negatively affect colonic motility and contribute to increased constipation in PD, given that butyrate increases colonic motility and stimulates neuronally driven contractility (Koh et al., 2016). Mechanistic evidence is provided by Sampson et al., who found that in germ-free and specific pathogen free wild-type as well as α-synuclein-overexpressing (ASO) mice, motor dysfunction and Parkinson’s disease (PD)-like features were notably enhanced in colonized (specific pathogen-free) and ASO mice. Fecal microbiota transfer from PD patients caused notably more PD features compared to microbiota transferred from controls; and this was particularly evident in ASO mice (Sampson et al., 2016).

The toll-like receptors (TLRs) which are innate immune receptors involved in pathogen recognition through conserved microbial patterns have been found to be involved in the immunopathology of PD due to the involvement in gut dysbiosis-linked inflammation and intestinal permeability; Toll receptor over-stimulation can activate enteric neuroglial circuit and α-synuclein protein aggregation (Dutta et al., 2019). Among thirteen mammalian toll-like receptors, ten TLRs have been found in human beings on immune cells (B cells, mast cells, NK cells, T regulatory cells, macrophages, monocytes, dendritic cells, neutrophils, basophils) and non-immune cells (epithelial cells, endothelial cells, neurons and glial cells in periphery and CNS (Brun et al., 2015; Préhaud et al., 2005). TLRs activate the transcription factors such as NF-κB, MAPK, and interferon-regulatory factor (IRF) pathways (Kawasaki and Kawai, 2014). Natively unfolded α-synuclein, which is a part of intrinsically disordered proteins under physiological conditions, tends to undergo pathological aggregation leading to the spectrum of synucleinopathies like PD (Spillantini et al., 1997). In an experimental model using mice, Fellner et al. observed that TLR4 directly binds to α-synuclein leading to microglial activation; deletion of TLR4 in mice leads to reduction in microglial clearance of α-synuclein leading to increased α-synuclein accumulation and degeneration of dopaminergic neurons in substantia nigra (Fellner et al., 2013). Other indications of gut dysbiosis in PD include decreased abundance of *Prevotella*, *Lactobacillus*, *Peptostreptococcus*, and *Butyricicoccus*, while there is an increase in populations of *Proteus* and *Enterobacter* in PD patients (Sampson et al., 2016). In progression of PD, dysfunctional enteric nervous system (ENS) causes dysautonomia and thus decreases GI motility leading to susceptibility to SIBO and disease burden (Wüllner et al., 2007). It was suggested by Bienenstock et al. that deficiency in SCFA, especially butyrate and propionate, deficiency may contribute to reduced GABA production and impaired neuroimmune signaling (Bienenstock et al., 2015). In a recent age- and sex-matched study conducted on 34 PD patients and 34 controls, showed significant changes in SCFA and gut microbiota profile in relation to increased GI dysmotility, impaired neurotransmitter synthesis and ENS dysfunction. Moreover, in animal studies it was found that SCFAs can modulate the expression of intestinal regulatory T Cells resulting in an increase in anti-inflammatory cytokines IL-10 and TGF-β which play important roles in neuro-immune communications (Unger et al., 2016; Smith et al., 2013). Although α-synuclein mouse models provide compelling mechanistic evidence for microbiota-mediated neuroinflammation, clinical studies have reported variability in the abundance of specific microbial taxa, likely reflecting differences in disease stage, geography, medication use, and analytical approaches.

### 4.3 Ocular diseases and the microbiota–mitochondria axis

Mounting evidence indicates that gut dysbiosis is involved in retinal pathologies via the interaction of metabolic and inflammatory pathways. The gut-retina axis refers to how gut microbiome affects the function of the retina, with gut dysbiosis being increasingly associated with various conditions such as diabetic retinopathy, age-related macular degeneration (AMD), retinitis pigmentosa, central serous chorioretinopathy (CSCR), retinal artery occlusion (RAO), and uveitis (Schiavone et al., 2025; Rowan and Taylor, 2018; Valdes et al., 2018). The gut microbiome may affect the response to stress hormones involved in the development of CSCR, thereby proposing a prophylaxis or treatment approach targeting gut microbiome (Schiavone et al., 2025). Gut dysbiosis has been associated with impaired intestinal barrier functions and thus increased bacterial translocation and immune activation; since the retina is immune-privileged and has low regenerative capabilities, it is highly susceptible to such chronic inflammation (Rowan and Taylor, 2018; Valdes et al., 2018).

#### 4.3.1 Age-related macular degeneration (AMD)

AMD, which is the main cause of irreversible blindness in elderly people, is becoming increasingly understood as a pathology that includes mitochondrial dysfunction, chronic inflammation, and metabolism disturbances. Retinal pigment epithelial (RPE) cells have extremely high mitochondrial density to provide for photoreceptors’ metabolism; thus, they are highly susceptible to oxidative damage and mtDNA damage. Age-related changes in mitochondria are responsible for impairment of oxidative phosphorylation, reduction of ATP generation, generation of mtROS, inflammasome activation, and finally lead to RPE cell degeneration and loss of photoreceptors. The recent data suggest that dysbiosis of the gut microbiota can exacerbate all these pathogenic processes via increasing intestinal permeability, endotoxemia, changes in bile acid metabolism, and decreased formation of SCFAs. Clinical studies revealed changes in the gut microbiota in AMD patients, characterized by the decrease in the number of bacteria producing SCFAs and enrichment with pro-inflammatory genera, which indicates the role of microbial dysbiosis in AMD development through the microbiota-mitochondria axis (Rowan and Taylor, 2018; Scuderi et al., 2022; Kaarniranta et al., 2019).

#### 4.3.2 Diabetic retinopathy (DR)

DR represents yet another retinal disease where mitochondrial dysfunction plays a key role in pathology. Chronic hyperglycemia leads to increased ROS production by mitochondria, mitochondrial DNA damage, endothelial dysfunction, inflammation via activation of NF-κB pathway and NLRP3 inflammasome, causing disruption of the blood-retinal barrier and gradual neurovascular degeneration of the retina. Recent studies show that changes in the gut microbiota play an important role in these processes by causing elevated levels of circulating lipopolysaccharides (LPS), intestinal barrier dysfunction, altered bile acids metabolism and decreased production of SCFAs. Helpful microbe genera like Akkermansia and Bifidobacterium seem to maintain intestinal barrier integrity and control mitochondrial bioenergetics, whereas proliferation of enterotoxigenic bacteria may increase oxidative stress and inflammatory response. Therefore, manipulation of the gut microbiome may be a way of indirect preservation of mitochondrial activity in the retina and slowing down progression of DR (Parker et al., 2020; Scuderi et al., 2022; Tang and Kern, 2011).

#### 4.3.3 Retinitis pigmentosa (RP)

RP is defined as a set of inherited retinal degenerative disorders caused by photoreceptor cell apoptosis. Genetic mutations trigger RP development, but mitochondrial dysfunction acts as an accelerating factor in this process due to oxidative phosphorylation failure, reduced ATP levels, increased formation of ROS, mitophagy disruption, and chronic neuroinflammation. According to recent preclinical research, changes in gut microbiota can affect retinal degeneration by influencing the functioning of the immune system, metabolism of microorganisms, and the oxidative stress mechanism. Although the direct link between gut dysbiosis and RP was not proved on the clinical level, these experimental data suggest that maintenance of mitochondrial homeostasis and normalization of gut microflora could become additional treatment options for the management of the disease. Nevertheless, further longitudinal and clinical investigations are needed to confirm the efficiency of these approaches in patients with RP (D’Amico et al., 2021; Scuderi et al., 2022; Campochiaro and Mir, 2018).

#### 4.3.4 Central serous chorioretinopathy (CSCR)

CSCR has been conventionally linked to psychological stress, glucocorticoid excess, and HPA axis imbalance. More recently, the role of gut microbiota in regulating the above pathways has emerged as a novel area of interest. Gut dysbiosis can lead to changes in metabolism of cortisol, systemic immunity, and gut permeability, resulting in a state of chronic low-grade inflammation that can affect the retinal pigment epithelium and choroidal blood flow. Despite the paucity of experimental data, these findings imply that microbiota-derived metabolites and mitochondrial dysfunction might play a role in disease development, which needs to be validated in clinical longitudinal studies (Cryan et al., 2019; Scuderi et al., 2022).

There is currently no clinical evidence to demonstrate the direct connection between gut dysbiosis and CSCR. Nevertheless, observational studies have shown that psychological stress, exposure to glucocorticoids, and HPA axis dysregulation play a crucial role in the onset of the disease.

Although mitochondrial dysfunction represents a common mechanism underlying retinal diseases, environmental factors may further accelerate these pathological processes. Among these, chronic exposure to artificial blue light has emerged as a potential contributor by disrupting circadian homeostasis, altering gut microbial composition, and promoting mitochondrial oxidative stress.

### 4.4 Environmental modifiers of the microbiota–mitochondria axis

**FIGURE 3:**
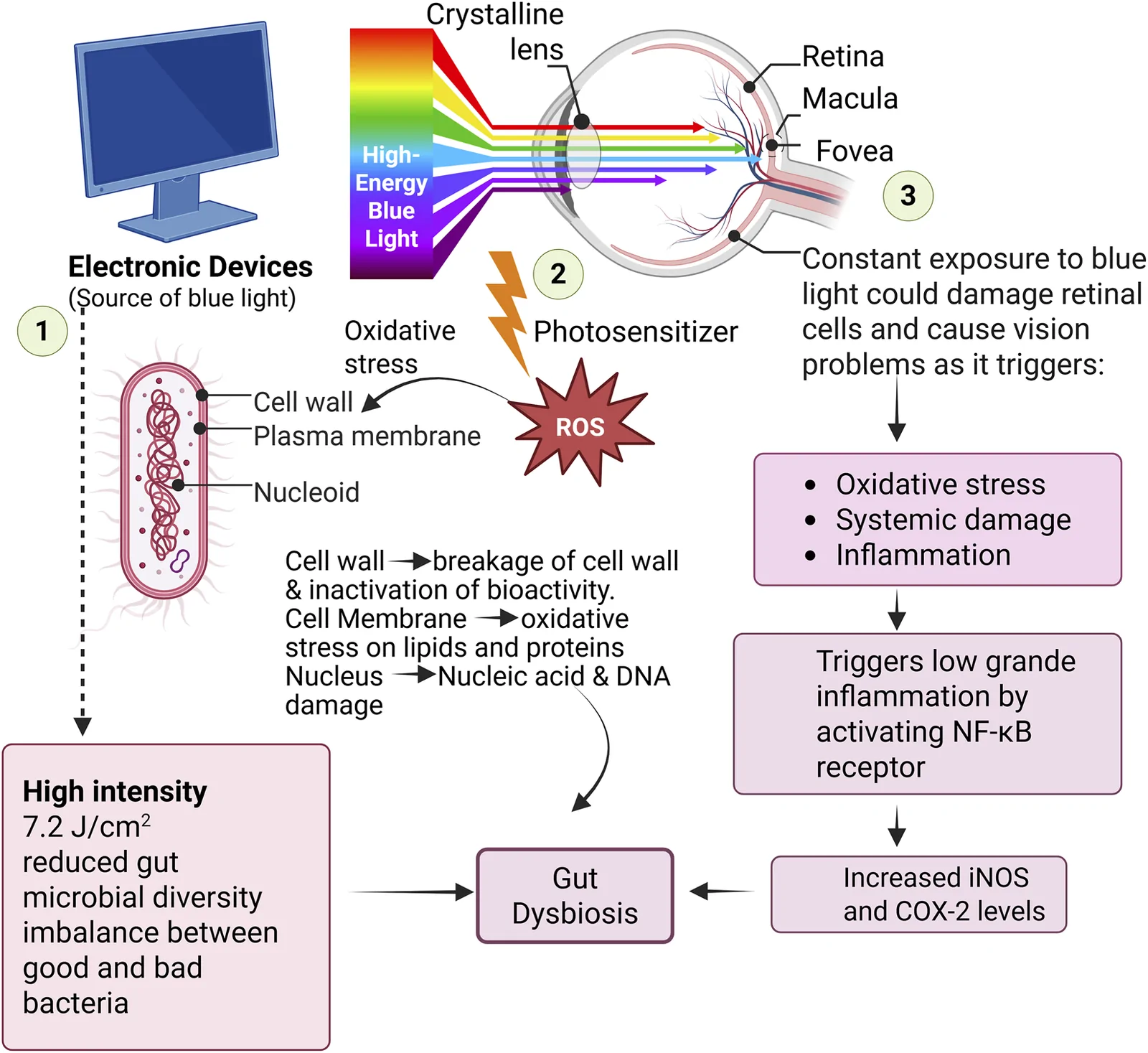
Mechanistic pathway linking blue light exposure to ocular damage and gut dysbiosis. Electronic devices act as a major source of high-energy blue light (7.2 J/cm^2^) that is able to reach the retina through the crystalline lens. High-energy blue light exposure leads to the disruption of photosensitizers’ cell wall and their bioactivity through the process of lipid and protein peroxidation as well as damage of nucleic acids/DNA in the cell wall and nucleus. All these processes initiate low-grade inflammation via the activation of the NF-κB signaling pathway and upregulation of iNOS and COX-2. The combination of these processes affects the retina in such a way that oxidative stress and low-grade inflammation occur, resulting in gut microbial dysbiosis.

Exposure to blue light is another important environmental factor contributing to the connection between eyesight and gastrointestinal health. Photosensitive ganglion cells of the retina, being most sensitive to blue light at wavelengths of 460–480 nm, signal through retinohypothalamic pathway to the suprachiasmatic nucleus (SCN), which inhibits activity of arylalkylamine N-acetyltransferase (AANAT) involved in melatonin production. This leads to the disturbance of circadian rhythm and sleep cycle (Duffy and Czeisler, 2009). Apart from sleep disorders, suppression of melatonin production, owing to its multiple functions in immune system regulation, DNA repair, endocrine function, and antioxidative protection, result in more widespread physiological imbalance causing metabolic and mental disorders (Wahl et al., 2019). At the same time, blue light triggers production of reactive oxygen species (ROS) in ocular tissues (cornea, lens, retina), causing oxidative damage related to eye strain, dry eye, cataract, retinal damage, and overall redox imbalance (Cougnard-Gregoire and Delcourt, 2023; Huang et al., 2023) (Figure 3).

The effect of lowered melatonin on compromised gut barrier function can be explained by degradation of tight junction proteins (occludin and claudin-1). This leads to increased intestinal permeability (“leaky gut”) and change towards pro-inflammatory microbial populations, which supports the gut-brain axis and the gut-eye-immune axis (Zhang et al., 2022; Anderson and Maes, 2012; Thaiss et al., 2014). Chronic blue light exposure has been reported to alter in abundance of Firmicutes (*Faecalibacterium*, *Ruminiclostridium_9*) and certain *Bacteroidetes* (*Bacteroides*, *Alistipes*, *Anaerofilum*), and decrease in *Ruminococcus*, *Dehalobacterium*, *Dorea*, *Anaeroplasma*, *Romboutsia*, and *Parabacteroides* (Zhang et al., 2022; Bonmatí-Carrión and Rol, 2023), although confirmation in human studies remains limited.

New genetics-based research has started to establish causality in research on diseases of the eye using genome-wide association studies, omics data sets, and Mendelian randomization techniques. For instance, there is evidence of causation of iridocyclitis due to type 1 diabetes from a large study involving genome-wide and omics data analysis; however, there is no such evidence for type 2 diabetes (Lu et al., 2025). This study additionally characterized molecular similarities between type 1 diabetes and iridocyclitis including pleiotropic genes like *PRCC* and *FUT2*, and gut microbiota similarity Parabacteroides, showing that particular microbial profiles could be involved in common metabolic and ocular inflammatory pathways (Lu et al., 2025). Importantly, while such genetically informed approaches provide stronger evidence, they still require mechanistic and interventional validation.

There is evidence of circadian rhythmicity in gut microbiota, dependent on the host circadian clock and feeding. These rhythms are necessary for the maintenance of metabolic homeostasis; any alteration in light/dark cycle or feeding rhythm leads to disruption of these cycles and increases chances for developing obesity, insulin resistance, and systemic inflammation (Thaiss et al., 2014). In the study conducted by Voigt et al., circadian disruption in mice (shift work/jet lag) led to loss of microbial rhythmicity, lowered diversity, and expansion of pro-inflammatory microbes. Transplantation of the dysbiotic microbiome to germ-free mice replicated the same results, confirming that circadian misalignment alone leads to dysbiosis and inflammation (Voigt et al., 2014).

Importantly, these studies were conducted in animal models under controlled laboratory conditions, and their applicability to human populations requires further clinical validation.

### 4.5 Nutritional modulation of the microbiota–mitochondria axis: The role of lutein

Lutein, a carotenoid from plants, is densely present in the retina of primates, especially in the macula that is the part of the retina involved in central vision, where lutein and zeaxanthin form the macular pigment important for retinal protection. There are two main mechanisms of protection of lutein: shielding of the blue light from damaging retinal structures and neutralizing free radicals through the action of an antioxidant. The exclusion of lutein from diet in animals causes fast retinal degeneration, while in people with macular telangiectasia, which is the loss of macular pigment, leads to serious visual acuity impairments (Wu et al., 2015).

The degree of prevention that the lutein consumption may play in reducing the occurrence of age-related macular degeneration (AMD) and cataracts, age-related vision problems, is still being actively researched. Lutein acts by binding with class B type 1 (SR-B1) and facilitates not only the absorption but also the transport of lutein into the retina and its subsequent distribution through the high-density lipoprotein (HDL) in the circulation. Polymorphism in genes encoding SR-B1 and HDL raises the AMD risk.

**FIGURE 4:**
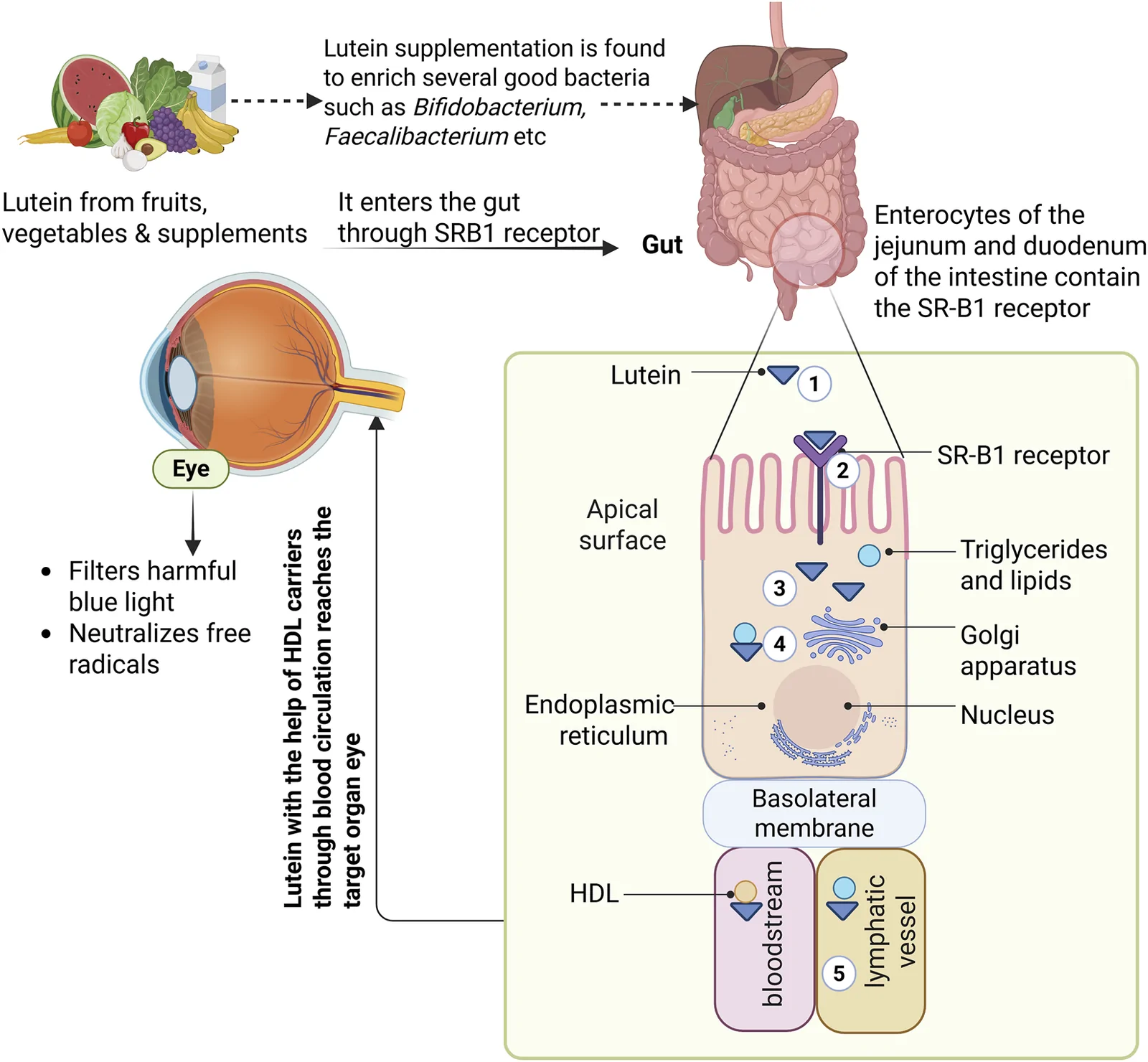
Gut absorption and ocular protection mechanism of lutein through the modulation of intestinal and retinal physiology. Intestinal lutein derived from dietary fruits, vegetables, and nutritional supplements gets absorbed into the body through the process of intestinal absorption mediated by the scavenger receptor class B type 1 (SR-B1). Intestinal absorption is followed by packaging of lutein within the chylomicrons with subsequent transport via cellular trafficking using Golgi bodies and endoplasmic reticulum to the basal region for release into the blood circulation. In the bloodstream, lutein binds to lipid molecules like high-density lipoprotein (HDL) and low-density lipoprotein (LDL). The build-up of lutein in the retina can help in filtering out harmful blue light as well as in scavenging for reactive oxygen species in order to prevent oxidative damage to the photoreceptors and retinal pigment epithelial cells. Moreover, it is possible that lutein supplementation can have positive effects on the gut microbiota, facilitating the growth of commensal bacteria like *Bifidobacterium* and *F*.*aecalibacterium*.

Besides its role in the vision system, lutein possesses anti-inflammatory properties, inhibiting NF-κB signaling along with iNOS and COX-2 expression (Scuderi et al., 2022). Since one of the main causes of AMD involves low-grade inflammatory response, this has triggered a study on whether the benefits of lutein are limited to the local protection and anti-inflammatory activity within the eye. Lutein, for instance, leads to beneficial modulation of microbiota populations, resulting in an increase in the presence of *Bifidobacterium*, *Faecalibacterium*, *Subdoligranulum*, and *Eubacterium ruminantium*, which are known to produce higher levels of short-chain fatty acids and reduce inflammation (Dinsmoor et al., 2019) (Figure 4). Most of the evidence regarding the association between gut dysbiosis, mitochondrial dysfunction, and retinal degeneration is derived from animal model studies, while human clinical trials are still lacking.

All the above findings suggested that mitochondrial dysfunction could be a critical pathway which mediates the effect of gut dysbiosis on neurodegeneration and retinal disorders. The disruption of neuronal and retinal homeostasis is associated by microbial metabolites, chronic inflammation, oxidative stress, and malfunction of mitochondrial quality control. As a result, the combination of treatment approaches that can normalize the gut microbiome and mitochondrial function becomes a promising avenue of disease treatment.

## 5 Mitochondria-targeted therapeutic strategies

### 5.1 Mitochondrial function modulators

The identification of mitochondrial dysfunctions as one of the major causes of neurodegenerative and ocular diseases has led to many attempts at creating therapies aimed at mitochondria (Majumdar et al., 2026b; Menna et al., 2026). Some of these drugs proved to be capable of enhancing mitochondrial activity and altering the intestinal microbiome composition and system-wide inflammation.

MitoQ is an antioxidant compound based on coenzyme Q10. It accumulates specifically in mitochondria and acts as a ROS scavenger. Experimental animal research on MitoQ has shown that it reduces neuroinflammation, increases mitochondrial respiration, preserves synapses and prevents cognitive impairment in neurodegeneration animal models (Murphy, 2009; Snow et al., 2010; Smith et al., 2011). MitoQ prevents oxidative stress-induced mitochondrial damage in retinal pigment epithelial (RPE) cells, preserves mitochondrial membrane potential, decreases ROS production and mitigates retinal degeneration in experimental models of AMD and DR (Kaarniranta et al., 2019; Menna et al., 2026; Kaštelan et al., 2026). These results are consistent with the therapeutic potential of MitoQ for ocular diseases, but clinical evidence is lacking and further randomized clinical trials are needed to establish its efficacy in patients with retinal disorders. However, clinical evidence supporting their efficacy in patients with neurodegenerative disorders remains limited.

SS-31 (Elamipretide) is a mitochondria-targeting tetrapeptide binding to cardiolipin and preserving the structural integrity of mitochondrial cristae. Experimental data suggest that SS-31 increases ATP biosynthesis, decreases oxidative stress and protects neurons against mitochondrial dysfunction-induced damage (Szeto, 2014). Elamipretide also showed promising protective effects in retinal degeneration by preserving photoreceptor mitochondrial integrity, reducing apoptosis and improving retinal function. Additionally, early phase clinical studies have assessed Elamipretide in patients with intermediate AMD, and have reported improvements in visual function, although larger randomized clinical trials are required to confirm long-term efficacy (Mettu et al., 2022; Allingham et al., 2022).

Coenzyme Q10 acts as an electron carrier of mitochondrial respiratory chain and is well studied in relation to PD and AD. Administration of coenzyme Q10 ameliorates mitochondrial bioenergetics and oxidative damage, but results remain controversial (Beal, 2003). Experimental data in ocular diseases suggest that coenzyme Q10 has a protective effect on retinal ganglion cells and retinal pigment epithelial cells against oxidative stress and mitochondrial dysfunction, indicating its potential therapeutic applications in glaucoma, diabetic retinopathy and age-related macular degeneration. But there is a lack of robust clinical evidence for its routine use in retinal disorders (Zhang et al., 2017; Russo et al., 2008; Wang et al., 2021).

Nicotinamide riboside (NR) and nicotinamide mononucleotide (NMN) replenish intracellular levels of NAD+ and promote mitochondrial repair via sirtuin signaling. Clinical and preclinical trials have demonstrated that NAD + supplementation promotes mitochondrial biogenesis, increases neuronal resistance and diminishes neuroinflammation (Lautrup et al., 2019). In addition, natural compounds, like resveratrol and spermidine, activate AMPK and autophagy signaling, thus increasing mitochondrial biogenesis and decreasing neuroinflammation (Madeo et al., 2018; Wu et al., 2011). NAD + precursors have been shown to preserve retinal ganglion cell survival, improve mitochondrial bioenergetics, reduce oxidative stress, and preserve retinal function in experimental models of retinal degeneration (Lin et al., 2016; Williams et al., 2017). However, these findings rely primarily on preclinical studies and further clinical trials are needed to assess their therapeutic potential in ocular diseases. Although, early clinical investigations have reported favorable safety profiles, robust evidence demonstrating clinical efficacy is still lacking.

Recently, urolithin A, a gut microbiota-produced metabolite from ellagitannins in the diet, has been found to induce mitophagy and improve mitochondrial biogenesis via the activation of the PINK1-Parkin pathway. As a result, urolithin A is considered to be a potential therapeutic agent for the treatment of neurodegenerative and retinal disorders (D’Amico et al., 2021; Ryu et al., 2016). Thus, these results indicate the therapeutic efficacy of an approach based on targeting both gut microbial homeostasis and mitochondrial function. Recent preclinical studies have also shown that urolithin A protects retinal pigment epithelial cells from oxidative injury, promotes mitophagy, preserves mitochondrial function and reduces retinal inflammation suggesting that modulation of the microbiota-mitochondria axis may be a promising therapeutic strategy for retinal degenerative diseases (Ryu et al., 2016; Andreux et al., 2019). However, clinical validation in ocular disorders is currently lacking. As mitochondrial dysfunction plays a critical role in both neurodegenerative and retinal disorders, much attention has been paid to therapies capable of regulating mitochondrial homeostasis and the composition of gut microbes simultaneously (Lautrup et al., 2019). There are several next-generation mitochondrial-targeting therapies that show promising results through microbiota-dependent actions.

### 5.2 Next-generation probiotics and postbiotics

Preclinical studies suggest that probiotics can improve mitochondrial function, attenuate neuroinflammation, and restore microbial homeostasis in experimental models of AD (Prajapati et al., 2025b; Prajapati et al., 2023). Similarly, preclinical evidence suggests that probiotic supplementation may protect against retinal oxidative stress, preserve mitochondrial function, and reduce retinal inflammation in experimental models of DR, AMD, and glaucoma by modulation of the gut–retina axis (Rowan et al., 2017; Schiavone et al., 2025; Kammoun et al., 2024).

The use of probiotics involves mostly species of *Lactobacillus* and *Bifidobacterium*. But the progress made so far includes the creation of the new generation of probiotics that are developed with the aim to influence host metabolism, inflammation, and mitochondria functioning. Potential new probiotics include *Akkermansia muciniphila*, *Faecalibacterium prausnitzii*, and microbial strains that can produce neuroprotective metabolites (Miquel et al., 2013; Depommier et al., 2019). However, clinical evidence demonstrating comparable therapeutic efficacy in humans remains limited, highlighting the need for well-designed randomized controlled trials.

Experimental animal studies demonstrate that postbiotics preserve mitochondrial integrity, reduce oxidative stress, and modulate inflammatory signaling. A postbiotic is considered to be any metabolite or product of microbial organisms that is used as a supplement. Short-chain fatty acids, especially butyrate, promote the process of oxidative phosphorylation, increase ATP formation, stimulate AMPK-PGC1α pathway, and inhibit neuroinflammation (Dalile et al., 2019; Silva et al., 2020). The usage of sustained release of butyrate for the therapy of neurodegenerative conditions that involve mitochondrial dysfunction is currently studied. In ocular diseases, preclinical studies have shown that butyrate and other microbiota-derived metabolites reduce retinal inflammation, preserve retinal pigment epithelial barrier function, improve mitochondrial respiration, and attenuate oxidative damage in models of DR and retinal degeneration (Dalile et al., 2019; Scuderi et al., 2022; Kaarniranta et al., 2020; Bringer et al., 2022). Although these findings are promising, clinical studies evaluating their therapeutic efficacy in neurodegenerative and ocular diseases are currently limited.

New technological solutions of synthetic biology make it possible to create the new strains of microorganisms that can provide the gut with mitochondrial nutrients, antioxidants, and anti-inflammatory metabolites (Charbonneau et al., 2020).

### 5.3 Dietary strategies targeting both microbiota and mitochondria

Modification of diet is a feasible method for the regulation of both gut microbiota composition and mitochondrial physiology. Of these, a ketogenic diet has become the focus of scientific investigations since ketone bodies can be used as another fuel for mitochondria and increase its efficiency (Newman and Verdin, 2017; D’Anniballe De Salles et al., 2026). The use of ketogenic interventions has been shown to increase mitochondrial biogenesis, decrease oxidative stress, improve neuronal energetics, and affect the composition of gut microorganisms in a positive way (Ang et al., 2020).

In a similar fashion, intermittent fasting and time-restricted feeding lead to stimulation of mitochondrial turnover and activation of autophagy and mitophagy pathways, better insulin sensitivity, and microbial diversity (Mattson et al., 2017). It was shown experimentally that intermittent fasting induces the synthesis of SCFAs by microbiota and at the same time activates the AMPK and sirtuin signaling pathways for the preservation of mitochondrial functions (De Cabo and Mattson, 2019).

Another interesting example of the gut microbiota-mitochondrial strategy is the Mediterranean diet. This diet contains plenty of dietary fiber, polyphenols, omega-3 fatty acids, and antioxidants; these features promote SCFA-producing bacteria, lower systemic inflammation, and benefit mitochondria (Trichopoulou et al., 2014; Ghosh et al., 2020). Collectively, these findings suggest that dietary interventions may exert dual therapeutic benefits through simultaneous modulation of gut microbial ecology and mitochondrial function. There is evidence from both preclinical studies and preliminary clinical trials suggesting that diet could have a positive effect on the gut microbiota composition, mitochondrial function, and systemic inflammation. At present, heterogeneity in clinical studies is substantial to make definitive treatment suggestions.

### 5.4 Personalized therapeutics based on metagenomics and metabolomics

Some of the promising directions in the future include personalized therapeutic approaches based on profiling of microbiome and metabolomics. The development of shotgun metagenomics sequencing, metabolomics, transcriptomics, and predictive modeling using artificial intelligence has made it possible to characterize microbial and metabolic markers of an individual precisely (Wishart, 2019; Integrative HMP, 2019).

The stratification of the patient is based on the microbial taxa, plasma SCFA, TMAO, mitochondrial biomarkers, and inflammation profile would aid in the precision of treatment choice. For instance, those individuals with lower SCFA producers will be helped with targeted postbiotic treatments, while people with high levels of TMAO may need changes in diet and microbial treatments to decrease TMA production (Vogt et al., 2018; Ge et al., 2025).

Precision medicine for the future may involve combining microbe-based therapeutic approaches with mitochondrial-based interventions for better efficacy. The combined approach could revolutionize the treatment of various diseases including AD, PD retinal degeneration, and other conditions linked with the dysfunction of the microbiota-mitochondria axis (Cryan et al., 2019; Fang et al., 2020).

Overall, the therapies that have been mentioned in this review such as probiotics, prebiotics, postbiotics, diet therapy, fecal microbiota transplantation, and mitochondria-targeted drugs showed promising effects mainly in cell culture studies and animal models. Despite the potential effects of certain interventions on patients reported in preliminary clinical trials, the existing clinical data are limited and inconsistent. Thus, these treatments can be viewed as promising but investigative and further longitudinal studies along with well-conducted RCTs will be required to prove their efficacy and safety in practice.

## 6 Strengths, limitations and evidence quality of current studies

### 6.1 Evidence synthesis and current knowledge gaps

Even though considerable progress has been achieved toward establishing an understanding of the microbiota-mitochondria axis in both neurodegenerative and retinal diseases, there is a marked disparity in the quality and robustness of the data obtained via different study designs. Biologically plausible associations between gut dysbiosis, mitochondrial dysfunction, and disease development have mostly been uncovered through *in vitro* studies, germ-free and antibiotic-pretreated animals, FMT experiments, and genetically engineered disease models (Cryan et al., 2019; Fang et al., 2020; Sampson et al., 2016; Harach et al., 2017). Direct translatability of such results into humans poses some difficulties due to the species differences in terms of gut microbiome, immune system, metabolism, life expectancy, and environment.

On the contrary, most of the studies conducted on humans are observational cohort studies where an association is observed between changes in gut microflora, levels of microbial metabolites, mitochondrial dysfunction, and disease severity (Cattaneo et al., 2017; Vogt et al., 2018). Although this research offers valuable clinical insgiht, still they cannot establish any causality and suffer from various confounding factors such as diet, medication use, age, genetics, lifestyle, and diseases. More recently, there has been an increase in the use of Mendelian randomization analysis and randomized controlled trials that have helped establish causality in relation to certain microbial species, microbial metabolites, and neurological disorders (Depommier et al., 2019; Ge et al., 2025). However, there have been some inconsistencies in the findings from such studies, mainly regarding microbial biomarkers and responses to therapy in relation to disease in various populations. The inconsistencies are due to disparities in sequencing technologies used, analysis techniques employed, host genetics, diet, drug intake, and disease status.

In summary, although there is a strong connection between gut dysbiosis, mitochondria dysfunction, and neurodegenerative/retinal disease, the exact causal pathways in humans have not yet been fully explored. Further studies using prospective cohort designs, multi-omics approaches, mechanistic clinical investigations, and randomized controlled trials with sufficient power are needed to confirm these mechanisms, to find reliable biomarkers, and to explore whether manipulation of the microbe-mitochondria axis can be utilized as a treatment option for such conditions. In order to give a more detailed account of the available evidence, the articles included in this review have been categorized according to study design and level of evidence (Table 2).

**TABLE 2:** evidence levels supporting the microbiota–mitochondria–brain axis.

Evidence type	Examples	Strengths	Limitations	Evidence level	References
Animal Models	Germ-free mice, APP/PS1 mice, α-synuclein mice, antibiotic-treated mice	Strong mechanistic insight	Limited human translation	Moderate	(Sampson et al., 2016; Harach et al., 2017)
*In vitro* Studies	Neuronal cultures, mitochondrial assays	Mechanistic precision	Lack systemic physiology	Moderate	(Pickles et al., 2018)
Human Cross-sectional Cohorts	AD, PD, AMD microbiome studies	Clinical relevance	Cannot establish causality	Moderate	(Cattaneo et al., 2017; Vogt et al., 2018)
Longitudinal Cohorts	Cognitive aging cohorts	Temporal associations	Residual confounding	Moderate–High	(Verhaar et al., 2022)
Mendelian Randomization Studies	Microbiome–AD analyses	Improved causal inference	Genetic instrument limitations	High	(Ge et al., 2025; Liang et al., 2025)
Randomized Controlled Trials	Probiotics, postbiotics, dietary interventions	Highest clinical evidence	Limited number available	High	(Depommier et al., 2019)
Multi-omics Human Studies	Metabolomics + microbiome + transcriptomics	Mechanistic human evidence	Expensive and limited scale	High	(Fang et al., 2020; Integrative HMP, 2019)

While there is significant progress achieved in terms of designing novel therapies that specifically target either the microbiota or the mitochondria, it is vital to realize that most of the current evidence comes from *in vitro* studies and animal models. The initial clinical studies that were conducted on the impact of dietary treatments, probiotics, and selected mitochondria-targeting drugs have shown promising safety profiles and efficacy; however, there is a lack of evidence provided by large-scale randomized controlled trials. Moreover, microbial diversity, heterogeneity of disease, host genetics, and various environmental factors may affect treatment efficacy and present important barriers to clinical translation.

## 7 Conclusion

Accumulating evidence suggests that gut dysbiosis and impaired mitochondrial function may act as common pathological mechanisms underlying neurodegenerative and ocular disorders, despite their distinct clinical manifestations. Gut dysbiosis regulates mitochondrial function through altered microbial metabolite production, immune activation, and disruption of mitochondrial homeostasis. Despite substantial advances in understanding the microbiota–mitochondria axis, much of the current mechanistic and therapeutic evidence remains preclinical. Consequently, the promising findings summarized in this review should be interpreted within the context of experimental models. Future longitudinal cohort studies, mechanistic investigations, and well-designed randomized clinical trials are required to validate these discoveries, establish causality, and determine whether microbiota- and mitochondria-targeted interventions can be safely and effectively translated into clinical practice for neurodegenerative and ocular diseases.
