# Supplementary material for: Microbiota-mitochondria axis in neurodegenerative disorders and retinal dysfunction: mechanisms and emerging therapeutic opportunities
Source: Front Pharmacol. 2026 Sep 1;17:1889898. doi: 10.3389/fphar.2026.1889898 (PMC13575618; doi:10.3389/fphar.2026.1889898)
Supplement: Supplementary file 1 [file Supplementaryfile1.docx]

**Supplementary Figure.1**

S.1. Blue light-induced disruption of the circadian rhythm and its impact on the gut–brain axis and microbiota composition. The above diagram represents the mechanistic pathway through which chronic exposure to blue light influences the circadian rhythm and the homeostasis of the gut. Exposure to blue light activates ipRGCs in the retina of the eye. The ipRGCs activate the master biological clock in the brain, the suprachiasmatic nucleus (SCN). The activation of the ipRGCs leads to the suppression of arylalkylamine N-acetyltransferase (AANAT) activity in the pineal gland. The suppression of AANAT activity reduces the synthesis of melatonin from tryptophan through serotonin. The decrease in melatonin production causes oxidative stress through increased ROS. The decrease in melatonin production disrupts the integrity of the epithelial cell layer of the gastrointestinal tract through altered tight junction protein expression, including occludin and claudin-1. The disruption of the epithelial cell layer leads to increased permeability of the gastrointestinal tract, also referred to as leaky gut. The disruption of the epithelial cell layer causes increased inflammation in the gastrointestinal tract through altered microbiota composition,

**Supplementary Figure .2**

S.2. The figure illustrates how intestinal dysbiosis may contribute to Parkinson’s disease. A reduction in short-chain fatty acids (SCFAs) in the gut disrupts intestinal barrier function by decreasing tight junction proteins (claudin-1 and claudin-2), leading to increased blood–brain barrier (BBB) permeability. This allows lipopolysaccharides (LPS) to trigger local inflammation, oxidative stress, and activation of microglia. The decline in SCFAs also causes dysfunction of microglia, activates the NLRP3 (NOD-like receptor protein 3) inflammasome, and enhances inducible nitric oxide synthase (iNOS) expression. Dysbiosis alters dopamine levels in the substantia nigra and changes secretion of neurotransmitters (NTs) such as dopamine and ghrelin. Bacterial amyloids may also mimic host proteins (molecular mimicry), leading to immune activation. Together, these processes promote neuroinflammation and degeneration of dopaminergic neurons, thereby worsening Parkinson’s disease pathology.

**Supplementary Figure.3**

S.3. Gut dysbiosis lowers SCFAs and increases TMAO; leaky gut allows LPS to enter blood and triggers inflammation, reactive oxygen species (ROS), and endothelial dysfunction, producing vascular stiffness, hypertension, and ultimately increased cardiovascular risk.
